# Rice Bran Consumption Improves Lipid Profiles: A Systematic Review and Meta-Analysis of Randomized Controlled Trials

**DOI:** 10.3390/nu17010114

**Published:** 2024-12-30

**Authors:** Soo-yeon Park, Yehyeon Kim, Min Ju Park, Ji Yeon Kim

**Affiliations:** Department of Food Science and Biotechnology, Seoul National University of Science and Technology, 232 Gongneung-ro, Nowon-gu, Seoul 01811, Republic of Korea

**Keywords:** rice bran, *Oryza sativa* L., lipid profiles, total cholesterol, triglycerides, meta-analysis

## Abstract

Background: Dyslipidemia, a leading risk factor for cardiovascular diseases (CVDs), significantly contributes to global morbidity and mortality. Rice bran, rich in bioactive compounds such as γ-oryzanol and tocotrienols, has demonstrated promising lipid-modulating effects. Objective: This meta-analysis aimed to evaluate the effects of rice bran on lipid profiles, including triglycerides (TG), total cholesterol (TC), low-density lipoprotein cholesterol (LDL-C), and high-density lipoprotein cholesterol (HDL-C), and identify factors influencing its efficacy across different populations and intervention conditions. Methods: A systematic search of PubMed, Web of Science, and Scopus was conducted to identify randomized controlled trials (RCTs) published up to November 2024. Effect sizes were calculated as mean differences with 95% confidence intervals (CIs). Subgroup analyses were performed based on intervention form, dosage, duration, region, and participant characteristics. Heterogeneity was estimated by the *I*^2^ statistic, and sensitivity analyses were conducted to assess the robustness of the findings. Results: Eleven RCTs involving 572 participants met the inclusion criteria. Pooled results showed that rice bran consumption significantly reduced TG (−15.13 mg/dL; 95% CI: −29.56, −0.71), TC (−11.80 mg/dL; 95% CI: −19.35, −4.25), and LDL-C (−15.11 mg/dL; 95% CI: −24.56, −5.66) with moderate heterogeneity (*I*^2^ = 38.1–63.0%). No significant changes were observed for HDL-C. Subgroup analyses showed that rice bran oil had greater effects on TC and LDL-C than whole rice bran. High-dose interventions (≥30 g/mL) and longer durations (>4 weeks) yielded stronger effects. Asian populations demonstrated greater reductions compared to Western populations. Conclusion: Rice bran, especially in the form of rice bran oil, significantly improves lipid profiles, supporting its role as a functional food for CVD prevention. Future research should focus on long-term studies with diverse populations to confirm its efficacy and explore underlying mechanisms.

## 1. Introduction

Cardiovascular disease (CVD) persists as the leading global cause of mortality, contributing significantly to both health challenges and economic burdens [1]. The rising prevalence of CVD has been strongly linked to modifiable risk factors, including poor dietary habits and physical inactivity, and metabolic imbalances, including dyslipidemia and chronic inflammation [2,3,4]. Dyslipidemia, defined by imbalances in blood lipid levels, including elevated levels of low-density lipoprotein (LDL) cholesterol and triglycerides, coupled with decreased high-density lipoprotein (HDL) cholesterol, is a key contributor to the development of CVD [5,6]. These lipid imbalances accelerate atherosclerosis, a condition where plaque buildup in arteries impairs blood flow, ultimately leading to heart attacks and strokes [7]. Addressing dyslipidemia is, therefore, a critical target in the prevention and management of CVD [8]. Dietary strategies have been identified as a primary approach for managing lipid profiles and reducing CVD risk [9]. Functional food ingredients with lipid-modulating and cardioprotective properties have gained significant attention in recent years [10,11,12]. Among these, rice bran—produced during rice milling—has been identified as a promising health-promoting ingredient due to its unique nutrient composition and bioactive properties [13,14].

Rice (*Oryza sativa* L.) is a dietary staple for over half of the world’s population, with white rice being the most commonly consumed form [15]. The production of white rice involves milling, a process that removes the bran and germ, leaving only the starchy endosperm [16]. Rice bran, a nutrient-dense by-product of this process, constitutes the outer layer of the rice grain and is typically discarded or repurposed despite its exceptional nutritional value. This layer is abundant in essential nutrients, including dietary fiber, vitamin E, and minerals, as well as a diverse range of bioactive compounds, including phenolics, tocotrienols, tocopherols, γ-oryzanol, and phytosterols [14,17,18]. Bioactive compounds, such as γ-oryzanol and tocotrienols, have been shown to support healthy blood lipid levels, alleviate oxidative stress, and reduce inflammation. These characteristics make rice bran a promising resource for the development of functional foods and dietary supplements [19,20,21].

Despite the potential health advantages of rice bran, much of the current research focuses on its isolated bioactive components or specific experimental contexts. There is a lack of comprehensive meta-analyses that systematically consolidate the evidence on the lipid-modulating effects of rice bran and its implications for cardiovascular and metabolic health. Given the increasing prevalence of dyslipidemia and the demand for dietary strategies that can effectively manage lipid profiles, a systematic evaluation of rice bran’s bioactive lipids is essential. Such an analysis would bridge the gap between isolated findings and provide a holistic understanding of how rice bran lipids influence lipid metabolism and related health outcomes. By synthesizing the current evidence, this study aims to evaluate the functional properties of rice bran lipids, elucidate their mechanisms of action in lipid regulation, and assess their overall impact on metabolic health. The findings from this study are anticipated to provide a foundation to inform future research and promote the application of rice bran lipids in the development of functional foods and dietary supplements targeting lipid-related disorders.

## 2. Materials and Methods

### 2.1. Data Sources and Search Strategies

A systematic literature search was conducted using PubMed, Web of Science, and Scopus, including studies published up to November 2024, without applying a specific starting date restriction. The objective was to identify clinical trials investigating the impact of rice bran supplementation on lipid profiles, specifically triglycerides (TGs), total cholesterol (TC), low-density lipoprotein cholesterol (LDL-C), and high-density lipoprotein cholesterol (HDL-C). The search keywords included: (“rice bran” OR “*Oryza sativa* bran” OR “*Oryza sativa* L. bran”) AND (“triglyceride” OR TG OR cholesterol OR “total cholesterol” OR TC OR “very low density lipoprotein” OR VLDL OR “low density lipoprotein” OR LDL OR “high density lipoprotein” OR HDL OR “apolipoprotein A” OR “apo A” OR “apolipoprotein B” OR “apo B” OR “lipoprotein” OR “lipid profile” OR lipid OR “cardiovascular disease” OR “heart disease” OR “hypercholesterolemia”).

The criteria for including studies in this systematic review were as follows: intervention studies conducted in humans evaluating the effects of oral rice bran supplementation on lipid profile improvement. The exclusion criteria included (i) studies unrelated to the specific material or functionality; (ii) in vitro or animal studies; (iii) studies lacking information on dosage and duration of supplementation; (iv) studies involving compound formulations (mixtures with other ingredients); (v) studies using inappropriate route of administration (e.g., topical application, inhalation); (vi) articles not presenting primary data (e.g., reviews); (vii) articles written in languages other than Korean or English.

To ensure a systematic and rigorous screening process for eligible articles, DistillerSR software (Ottawa, ON, Canada) was used to manage the review workflow, including title and abstract screening, full-text assessment, and data extraction. The initial screening of title and abstract screening (Level 1) was independently conducted by two reviewers using the software’s dual reviewer screening feature. Articles meeting the inclusion criteria by both reviewers were advanced to full-text screening (Level 2). Any disagreements during the screening process were resolved through discussion and consensus, with another reviewer consulted as needed.

During the full-text screening phase, both reviewers independently assessed the studies, with conflicts resolved through the same consensus process. DistillerSR facilitated key tasks, including conflict resolution, documentation of exclusion reasons, duplicate detection, and management of data from multiple databases. Additionally, the software supported risk of bias assessments and generated reporting templates following the Preferred Reporting Items for Systematic Reviews and Meta-Analyses (PRISMA) guidelines to ensure a rigorous and transparent review process.

### 2.2. Selection Criteria

The inclusion criteria were established based on the PICO (Population, Intervention, Comparison, Outcome) framework and were defined as follows:

1. Population: individuals who are healthy, exhibit borderline cholesterol abnormalities, or are diagnosed with hypercholesterolemia
2. Intervention: clinical trials investigating the impact of rice bran or its extracts
3. Comparison: use of a placebo as the comparator
4. Outcome: randomized controlled trials employing either a parallel or crossover design

### 2.3. Data Extraction, Quality Assessment, and Publication Bias

Two researchers independently reviewed the articles that passed the initial screening phase. The following data were extracted from eligible articles: (i) the primary author’s name; (ii) year of publication; (iii) type of material studied; (iv) details of the RCT design; (v) participant characteristics; (vi) sample size; (vii) intervention duration; and (viii) relevant information for meta-analysis.

The quality of each selected study was evaluated using the Cochrane Risk of Bias (ROB) assessment tool, which evaluates potential biases across the following five domains: (i) bias arising from the randomization process; (ii) bias due to deviations from intended interventions or non-adherence to intervention assignments; (iii) bias resulting from incomplete outcome data; (iv) bias in measurement of the outcome; (v) bias in the selective reporting of results. Each domain was classified as having either a “low risk of bias”, a “high risk of bias”, or an “unclear risk of bias” [22]. The risk of bias evaluation relied exclusively on information provided in the published literature. In addition, study quality was comprehensively assessed, considering factors such as data quality, quantity, and consistency. The findings were reviewed in an integrated manner to provide a comprehensive evaluation of the functional evidence.

### 2.4. Statistical Analysis

The overall effect size of rice bran supplementation on lipid profiles was calculated according to the methods outlined in the Cochrane Handbook [23]. The effect size for each study was combined using the mean difference in the changes observed for blood lipid levels. Mean values and standard deviations (SD) before and after the intervention were extracted for both the rice bran consumption group and the placebo group, and the mean change was calculated relative to the placebo group.

In cases of high heterogeneity between studies, the random-effects model with the Dersimonian and Laird weighting method [24] was applied to evaluate the overall effect size. Heterogeneity among the included studies was assessed using the *Q*-test [25] and the *I*^2^ statistic [26]. An *I*^2^ value of less than 25% indicated low heterogeneity, 25–75% indicated moderate heterogeneity, and greater than 75% indicated high heterogeneity. To evaluate publication bias, a funnel plot was visually inspected to assess symmetry, and Egger’s regression test was used to determine statistical significance. To assess the robustness of the results, a sensitivity analysis was conducted using a one-study omitted analysis approach.

Statistical analyses were performed using Comprehensive Meta-Analysis (CMA) version 4.0 (Biostat, Englewood, NJ, USA). The results included effect sizes with corresponding 95% confidence intervals (CIs), and statistical significance was determined at a *p*-value of less than 0.05.

## 3. Results

### 3.1. Study Selection, Risk of Bias Assessment, and Characteristics of Included Studies

**Figure 1:**
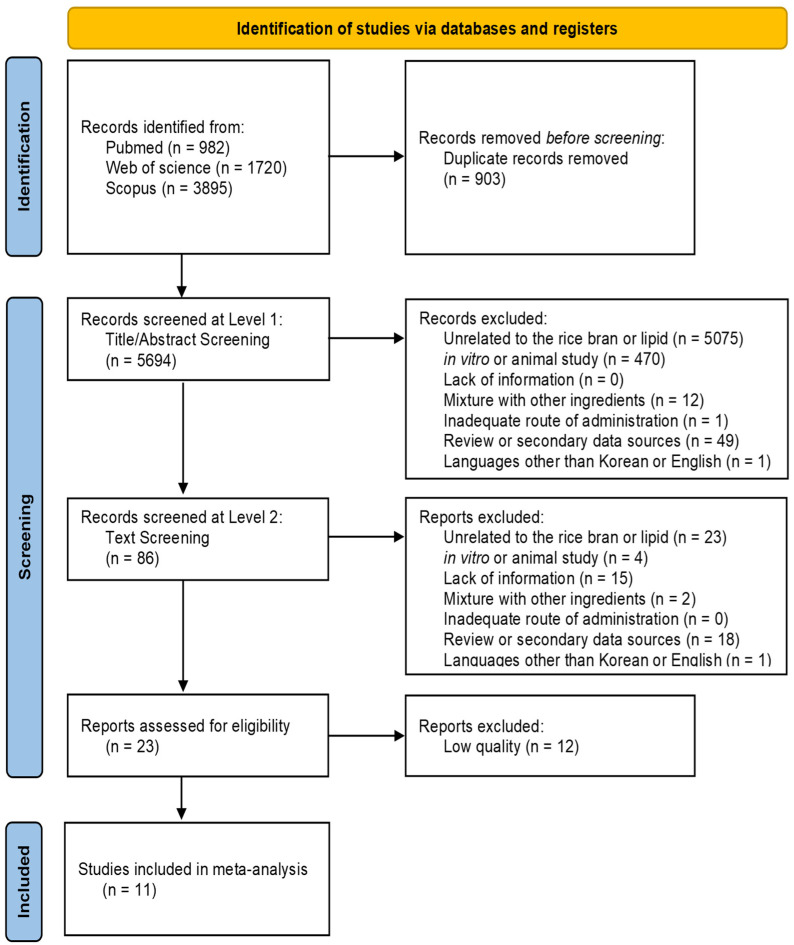
PRISMA flow diagram for literature search and study selection.

A total of 6597 records were identified through database searches, with 982 from PubMed, 1720 from Web of Science, and 3895 from Scopus. After removing 903 duplicate records, 5694 unique records were screened in the initial title and abstract screening phase (Level 1). During the Level 1 screening, 5608 records were excluded for the following reasons: 5075 records were unrelated to rice bran or lipid-related outcomes; 470 were in vitro or animal studies; 12 assessed rice bran as part of a mixture with other ingredients; one used an inadequate route of administration; 49 were review articles or data sources, and one was published in a language other than Korean or English. As a result, 86 records proceeded to full-text screening (Level 2). During the Level 2 screening, 63 reports were excluded based on the inclusion and exclusion criteria, leaving 23 reports for eligibility assessment. A thorough quality assessment of these studies led to the exclusion of an additional 12 studies due to low quality. Ultimately, 11 studies were included in the final meta-analysis. This multi-step screening and selection process is summarized in Figure 1, following PRISMA guidelines.

**Figure 2:**
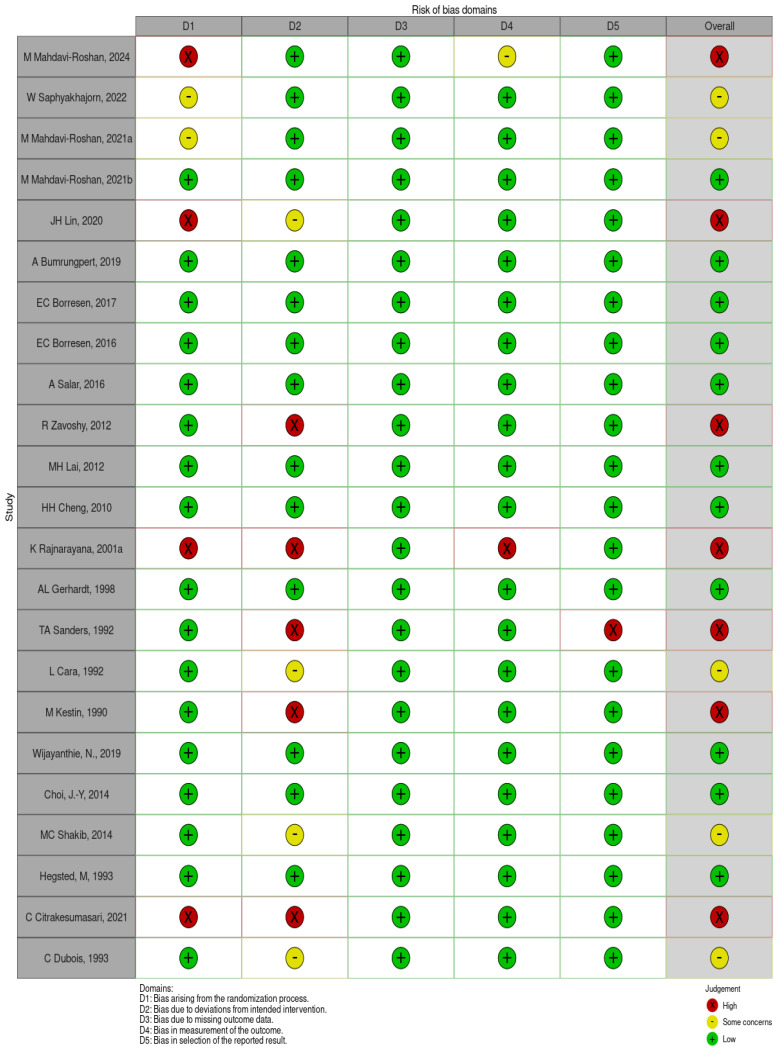
Risk of bias assessment for each included study [27,28,29,30,31,32,33,34,35,36,37,38,39,40,41,42,43,44,45,46,47,48,49].

A total of 23 studies were included, and the risk of bias assessment identified that 11 studies [27,28,29,30,31,32,33,34,35,36,37] were classified as “low risk of bias”, five studies [38,39,40,41,42] as having “some concerns”, and seven studies [43,44,45,46,47,48,49] as “high risk of bias” (Figure 2). The 11 low-risk studies met various evaluation criteria, including randomization, double-blinding, baseline group balance, adequacy of statistical analysis or biomarker assessment, and dropout rates (or any adverse events impacting participants’ health status). These factors supported their classification as studies with a low risk of bias.

The characteristics of the 11 randomized controlled trials (RCTs) included in the meta-analysis are summarized in Table 1. These studies investigated various interventions, including rice bran oil (five studies), rice bran (five studies), and fermented rice bran (one study). The interventions were administered in different forms and dosages, ranging from 3 g to 100 g or 15 mL to 30 mL per day, depending on the study design and participant characteristics.

### 3.2. The Effect of Rice Bran Consumption on Blood Lipid Profiles

**Figure 3:**
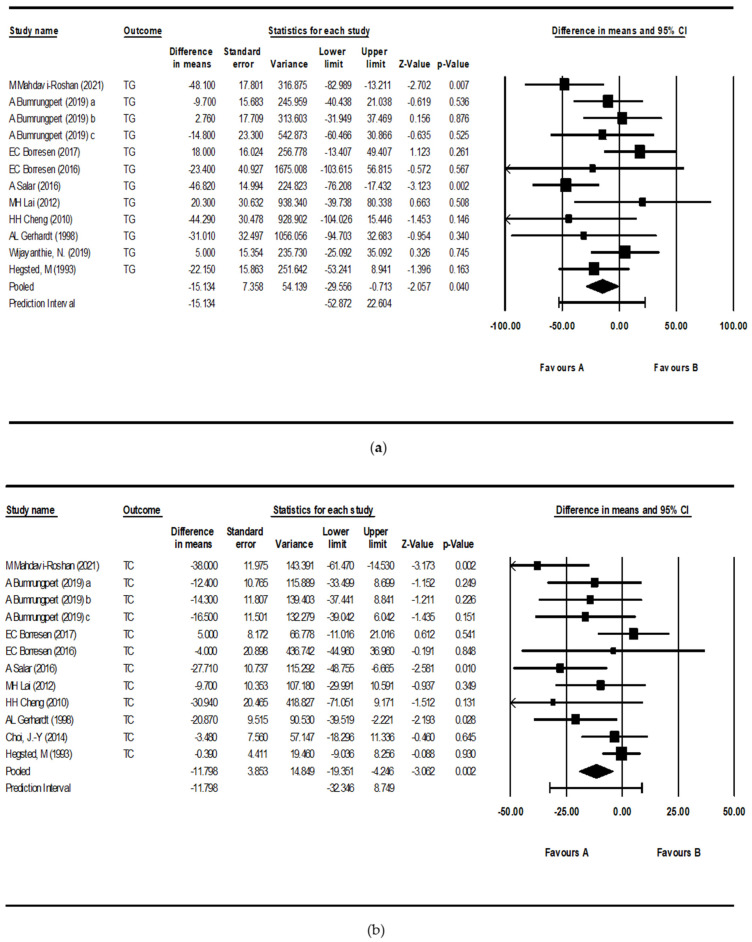

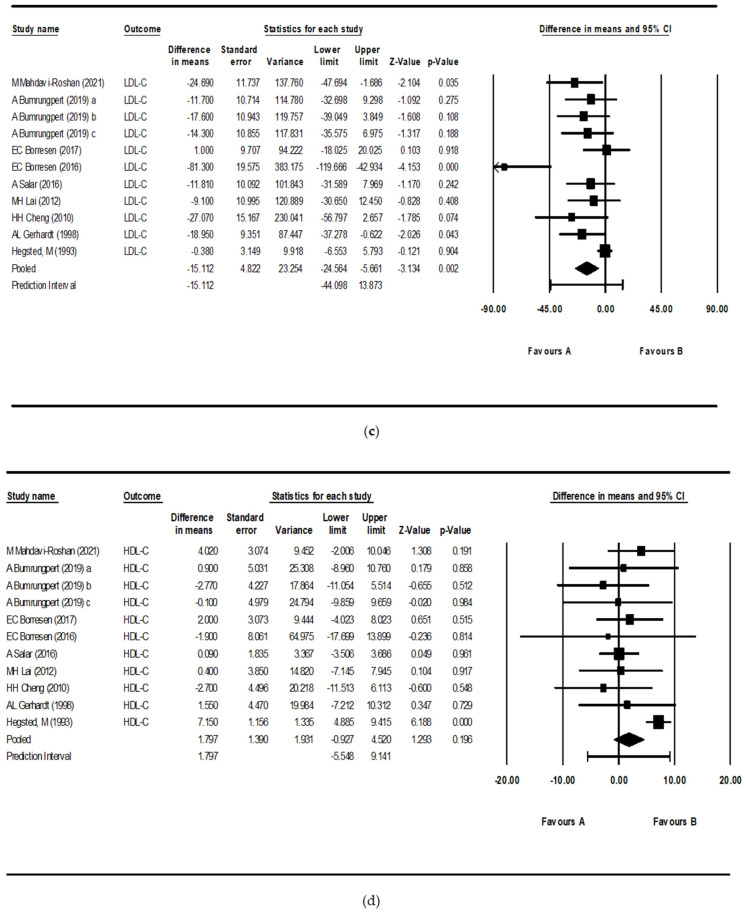
Forest plots of the effect of rice bran on (**a**) triglycerides (TGs), (**b**) total cholesterol (TC), (**c**) low-density lipoprotein cholesterol (LDL-C), and (**d**) high-density lipoprotein cholesterol (HDL-C). Each square represents the point estimate of an individual study, with its size reflecting the study’s weight in the meta-analysis (based on sample size and precision). Squares are shown with 95% confidence intervals (CIs), and the diamond represents the pooled mean difference with its 95% CI. ^a, b, c^ represent rice bran containing different levels of gamma-oryzanol: a = 4000 ppm, b = 8000 ppm, c = 11,000 ppm [27,28,29,30,31,32,33,34,35,36,37].

Rice bran intake significantly reduced TG levels compared to the placebo group by −15.13 mg/dL (95% CI: −29.56, −0.71; *p* = 0.040), with moderate heterogeneity observed (*I*^2^ = 38.1%; *p* = 0.087). Similarly, TC levels were significantly reduced by −11.80 mg/dL (95% CI: −19.35, −4.25; *p* = 0.002) compared to the placebo group, with moderate heterogeneity also observed (*I*^2^ = 44.0%; *p* = 0.051). LDL-C levels were also significantly reduced compared to the placebo group by −15.11 mg/dL (95% CI: −24.56, −5.66; *p* = 0.002), with moderate heterogeneity (*I*^2^ = 63.0%; *p* = 0.003). However, rice bran intake resulted in no significant change in HDL-C levels compared to the placebo group (1.80 mg/dL; 95% CI: −0.93, 4.52; *p* = 0.196), despite moderate heterogeneity (*I*^2^ = 49.5%; *p* = 0.031) (Figure 3).

### 3.3. Publication Bias

**Figure 4:**
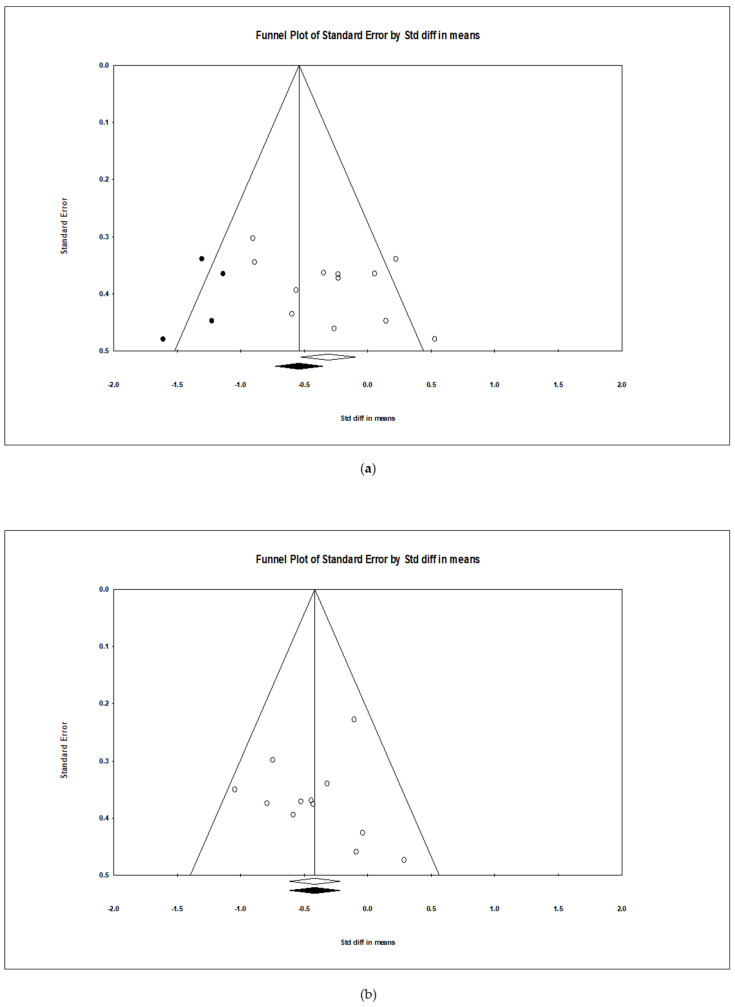

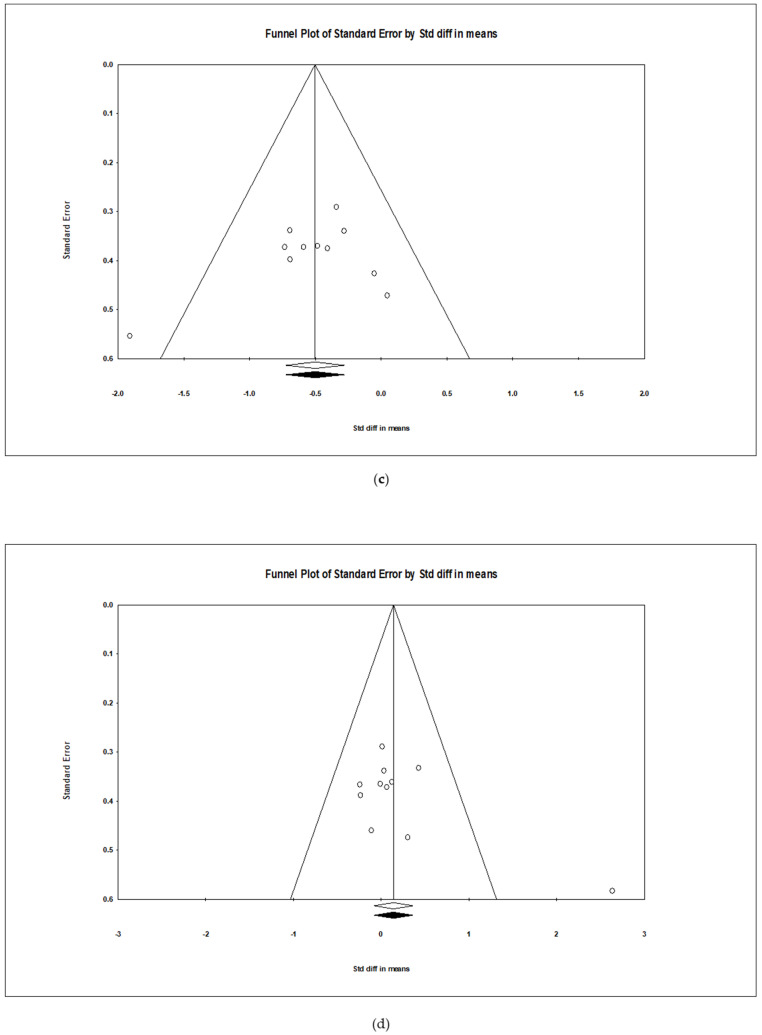
Funnel plots of studies included in this meta-analysis showing the effects of rice bran intake on (**a**) triglycerides (TGs), (**b**) total cholesterol (TC), (**c**) low-density lipoprotein cholesterol (LDL-C), and (**d**) high-density lipoprotein cholesterol (HDL-C). Funnel plot of standard error (y-axis) versus standardized mean difference in means (x-axis). Each circle represents an individual study included in the meta-analysis.

The studies evaluating the effect of rice bran intake on blood lipid level improvement showed a typical funnel plot shape. Egger’s regression analysis confirmed that publication bias was not statistically significant for triglycerides (TGs; *p* = 0.112), total cholesterol (TC; *p* = 0.967), low-density lipoprotein cholesterol (LDL-C; *p* = 0.248), and high-density lipoprotein cholesterol (HDL-C; *p* = 0.058) (Figure 4).

### 3.4. Sensitivity Analysis

To evaluate the influence of individual studies on the overall effect size, a sensitivity analysis was performed by sequentially excluding each study from the meta-analysis. For TG and LDL-C, the exclusion of individual studies did not significantly impact the overall effect size. However, for TGs, the exclusion of the studies by Bumrungpert et al. [28], Borresen et al. [30], Cheng et al. [33], Gerhardt et al. [34], and Hegsted et al. [37] resulted in a weakened effect size with a decreasing trend (0.05 < *p* < 0.1). Notably, the exclusion of Mahdavi-Roshan et al. [27] resulted in the loss of statistical significance (*p* > 0.1). The results of the omitting one study analysis, as shown in Table 2, reveal that negative values of the effect size for TGs, TC, and LDL-C reflect reductions in these parameters, while positive values for HDL-C reflect increases in HDL-C levels.

### 3.5. Subgroup Analysis

Subgroup analyses revealed significant variations in the effects of interventions on total cholesterol (TC) and low-density lipoprotein cholesterol (LDL-C) based on study design, health status of subjects, intake form, dosage, duration, region, gender, and age (Table 3).

For study design, parallel studies significantly reduced TC (effect size: −13.86; 95% CI: −21.71, −6.02), with moderate heterogeneity (*I*^2^ = 31.1%), while crossover studies showed no significant effect (effect size: −0.39; 95% CI: −9.04, 8.26; *I*^2^ = 0.00%). Similarly, LDL-C was significantly reduced in parallel studies (effect size: −17.42; 95% CI: −26.88, −7.96) with moderate heterogeneity (*I*^2^ = 44.3%), whereas crossover studies showed no significant reduction (effect size: −0.38; 95% CI: −6.55, 5.79; *I*^2^ = 0.00%).

In terms of health status, reductions in TC were observed in the hyperlipidemia subgroup (effect size: −7.12; 95% CI: −15.54, −1.30; *I*^2^ = 33.0%), with no heterogeneity. For LDL-C, reductions were found in the hyperlipidemia subgroup (effect size: −7.25; 95% CI: −14.97, −0.47), with low heterogeneity (*I*^2^ = 29.6%). The T2DM subgroup showed significant reductions in TC (effect size: −19.85; 95% CI: −33.57, −6.12), while no significant reductions were observed in LDL-C (effect size: −13.77; 95% CI: −26.85, −0.68), both with no heterogeneity.

The type of intervention also influenced the outcomes. Rice bran oil (RBO) significantly reduced both TC (effect size: −19.24; 95% CI: −28.16, −10.33) and LDL-C (effect size: −14.55; 95% CI: −23.24, −5.87), with no heterogeneity (*I*^2^ = 0.00%). Rice bran showed reductions in TC (effect size: −5.65; 95% CI: −16.70, −5.41) and LDL-C (effect size: −19.34; 95% CI: −38.86, 0.18), but these reductions were not statistically significant.

For dose, high-dose interventions (≥30 g/mL) resulted in significant reductions in both TC (effect size: −15.94; 95% CI: −26.22, −5.67) with moderate heterogeneity (*I*^2^ = 52.7%) and LDL-C (effect size: −17.72; 95% CI: −29.86, −5.59) with substantial heterogeneity (*I*^2^ = 71.5%). Low-dose interventions (<30 g/mL) did not result in significant reductions in either TC or LDL-C, and heterogeneity remained low (*I*^2^ = 7.35 for TC; *I*^2^ = 18.4% for LDL-C).

Intervention duration also played a role. Longer durations (>4 weeks) significantly reduced TC (effect size: −17.45; 95% CI: −30.28, −4.62; *I*^2^ = 44.9%) and LDL-C (effect size: −18.81; 95% CI: −29.88, −7.73; *I*^2^ = 0.00%), with moderate to no heterogeneity. Shorter durations (≤4 weeks) showed no significant reductions for TC but exhibited substantial heterogeneity (*I*^2^ = 34.4%), whereas LDL-C reductions were smaller (effect size: −13.70; 95% CI: −26.42, −0.98) with high heterogeneity (*I*^2^ = 70.9%).

Regionally, studies conducted in Asia showed significant reductions in TC (effect size: −16.24; 95% CI: −24.56, −7.91; *I*^2^ = 16.0%), with low heterogeneity, and LDL-C (effect size: −15.54; 95% CI: −23.88, −7.20; *I*^2^ = 0.00%), with no heterogeneity. Studies from the USA showed smaller and non-significant reductions in TC (effect size: −3.61; 95% CI: −14.11, 6.90; *I*^2^ = 37.5%) and LDL-C (effect size: −18.11; 95% CI: −40.17, 3.95; *I*^2^ = 84.8%), with moderate to high heterogeneity.

Gender analysis revealed significant reductions in TC in studies with mixed-gender participants (effect size: −5.86; 95% CI: −11.43, −0.30; *I*^2^ = 2.89%), indicating minimal heterogeneity. Male-only studies showed larger reductions in TC (effect size: −38.00; 95% CI: −61.47, −14.53) and LDL-C (effect size: −24.69; 95% CI: −47.69, −1.69; *I*^2^ = 0.00%), both with no heterogeneity.

For age, participants aged ≥18 years showed significant reductions in TC (effect size: −13.67; 95% CI: −21.45, −5.89; *I*^2^ = 39.8%) and LDL-C (effect size: −17.16; 95% CI: −27.53, −6.79; *I*^2^ = 65.7%), with moderate heterogeneity. No significant changes were observed for TC and LDL-C in participants aged <18 years.

## 4. Discussion

This meta-analysis demonstrates that rice bran consumption, particularly in the form of rice bran oil (RBO), significantly reduces total cholesterol (TC), low-density lipoprotein cholesterol (LDL-C), and triglycerides (TGs), reinforcing its potential as a functional food for cardiovascular disease prevention. The findings align with previous studies that have established the role of bioactive compounds, such as γ-oryzanol, tocotrienols, and phytosterols, in modulating lipid metabolism through multiple mechanisms [50,51]. These include inhibition of cholesterol absorption, reduction of hepatic cholesterol synthesis, enhancement of LDL receptor activity, and promotion of bile acid excretion [52]. Specifically, γ-oryzanol and tocotrienols, highly concentrated in RBO, act as potent inhibitors of HMG-CoA reductase, reducing endogenous cholesterol synthesis while also exerting antioxidant and anti-inflammatory effects that further support cardiovascular health [53,54].

The subgroup analyses revealed key factors influencing the efficacy of rice bran interventions. High doses (≥30 g/mL) and longer durations (>4 weeks) were associated with greater reductions in TC and LDL-C, underscoring the importance of intervention intensity and adherence. Sustained intake likely allows for the cumulative effects of bioactive compounds on lipid metabolism, while higher doses may exceed a threshold necessary to achieve meaningful changes in lipid profiles. These findings are consistent with the dose-response relationships observed in similar dietary interventions.

Regional variations in the lipid-lowering effects were evident, with studies conducted in Asia showing stronger effects than those in Western populations. This may be attributed to baseline dietary patterns, genetic predispositions, or differences in rice bran preparation methods. Asian populations often consume diets rich in rice and low in saturated fats, which may synergize with rice bran to amplify its lipid-lowering effects. Additionally, genetic polymorphisms in lipid metabolism-related genes, such as APOE and LDLR, could contribute to enhanced responsiveness among Asian participants [55,56].

Gender-specific and age-related differences were also noted. Male participants demonstrated larger reductions in TC and LDL-C compared to females, suggesting potential sex-based variations in lipid metabolism and response to rice bran. These differences may be influenced by hormonal regulation and hepatic LDL receptor activity [57,58]. In terms of age, participants aged ≥18 years showed significant lipid reductions, while no significant changes were observed in younger populations, possibly due to differences in baseline lipid levels or dietary behaviors.

Publication bias was not detected in this meta-analysis, which strengthens the validity of the findings. Sensitivity analyses further confirmed the robustness of the results, as the exclusion of individual studies did not substantially alter the pooled effect sizes for TC and LDL-C. However, studies with longer durations and higher doses appeared to have a stronger influence on the overall effect, highlighting the importance of these factors in the design of effective dietary interventions.

Despite the promising results, this meta-analysis has several limitations. First, regional variations in lipid-lowering effects were evident, particularly in studies conducted in Western populations. For example, reductions in LDL-C showed higher heterogeneity, potentially due to differences in dietary patterns, genetic predispositions, and variability in intervention protocols. These inconsistencies limit the robustness of the findings when generalized to Western populations. Second, the small number of studies in certain subgroups, such as younger participants (<18 years) and specific intervention forms (e.g., fermented rice bran), reduces the statistical power to detect significant subgroup differences. This limits the ability to generalize the findings across diverse demographic and intervention contexts. Third, variability in study durations and follow-up periods poses another limitation. While longer interventions demonstrated stronger effects, the lack of long-term follow-up data hinders understanding of the sustainability of rice bran’s lipid-modulating effects over time.

To address these issues, future research should prioritize well-designed randomized controlled trials with standardized protocols to minimize heterogeneity. Including studies from more diverse populations and exploring the effects of rice bran across various demographic groups, health conditions, and intervention forms will enhance the generalizability of the findings. Additionally, long-term studies are needed to evaluate the durability of rice bran’s lipid-lowering effects.

These results emphasize the potential of rice bran as a practical and accessible dietary strategy for improving lipid profiles and preventing CVD, particularly when tailored to specific population needs. With growing global interest in plant-based, functional foods, rice bran represents a sustainable, cost-effective option for public health interventions targeting dyslipidemia [15]. Rice bran could be incorporated into dietary recommendations, functional food development, and public health campaigns to reduce the burden of CVD [59]. For example, rice bran oil-based cooking oils or fortified foods could serve as practical tools for individuals at high risk of dyslipidemia [60]. Moreover, leveraging rice bran as a by-product of rice milling aligns with global sustainability goals, providing a low-cost resource that is widely accessible, particularly in rice-consuming regions [61]. To maximize its impact, future studies should focus on refining its applications, such as optimizing bioavailability and developing culturally tailored products, while expanding research to underserved populations to ensure equitable health benefits globally.

## 5. Conclusions

In conclusion, rice bran consumption, particularly in the form of RBO, significantly improves blood lipid profiles, with notable reductions in TC and LDL-C. Subgroup analyses underscore the importance of factors such as health status, dosage, duration, and intervention form in modulating these effects. These findings provide a strong evidence base for the use of rice bran as a functional food for cardiovascular health while also highlighting the need for further research to optimize its application across diverse populations.

## Figures and Tables

**Table 1:** Characteristics of clinical studies included in the meta-analysis.

Study Name	Study Design	Intervention	Country	Subject	Sample Size	Dose (/Day)	Duration (Weeks)	Outcomes
M Mahdavi-Roshan (2021), [27]	RCT, P	Rice bran oil	Iran	CAD	40	30 g	8	TGs, TC, LDL-C, HDL-C
A Bumrungpert (2019) ^a^, [28]	RCT, DB, P	Rice bran oil	Thailand	Hyperlipidemia	14	30 mL	4	TGs, TC, LDL-C, HDL-C
A Bumrungpert (2019) ^b^, [28]	RCT, DB, P	Rice bran oil	Thailand	Hyperlipidemia	15	30 mL	4	TGs, TC, LDL-C, HDL-C
A Bumrungpert (2019) ^c^, [28]	RCT, DB, P	Rice bran oil	Thailand	Hyperlipidemia	15	30 mL	4	TGs, TC, LDL-C, HDL-C
EC Borresen (2017), [29]	RCT, P	Rice bran	USA	Hyperlipidemia	50	15 g	4	TGs, TC, LDL-C, HDL-C
EC Borresen (2016), [30]	RCT, SB, P	Rice bran	USA	CRC	37	30 g	4	TGs, TC, LDL-C, HDL-C
A Salar (2016), [31]	RCT, SB, P	Rice bran oil	Iran	T2DM	75	30 g	4	TGs, TC, LDL-C, HDL-C
MH Lai (2012), [32]	RCT, SB, P	Rice bran oil	Taiwan	T2DM	40	18 g	5	TGs, TC, LDL-C, HDL-C
HH Cheng (2010), [33]	RCT, DB, P	Rice bran	Taiwan	T2DM	28	20 g	12	TGs, TC, LDL-C, HDL-C
AL Gerhardt (1998), [34]	RCT, DB, P	Rice bran	USA	Hyperlipidemia	52	84 g	6	TGs, TC, LDL-C, HDL-C
Wijayanthie, N. (2019), [35]	RCT, SB, CO	Rice bran oil	Indonesia	T2DM	10	15 mL	4	TGs
Choi, J.-Y (2014), [36]	RCT, DB, P	Fermented rice bran	South Korea	Healthy	80	3 g	8	TC
Hegsted, M (1993), [37]	RCT, CO	Rice bran	USA	Hyperlipidemia	11	100 g	3	TG, TC, LDL-C, HDL-C

^a^, ^b^, and ^c^ represent studies that investigated interventions with gamma-oryzanol at concentrations of 4000 ppm, 8000 ppm, and 11,000 ppm, respectively. RCT, Randomized Controlled Trial; DB, Double-Blind; SB, Single-Blind; P, Parallel; CO: Crossover; CAD, Coronary Artery Disease; T2DM, Type 2 Diabetes Mellitus; CRC, Colorectal Cancer; TGs, Triglycerides; TC, Total Cholesterol; LDL-C, Low-Density Lipoprotein Cholesterol; HDL-C, High-Density Lipoprotein Cholesterol.

**Table 2:** Results of the omitting one study analysis.

Omitted Study	TGs			TC			LDL-C			HDL-C		
	Effect Size	95% CI	*p*-Value	Effect Size	95% CI	*p*-Value	Effect Size	95% CI	*p*-Value	Effect Size	95% CI	*p*-Value
Mahdavi-Roshan, M (2021), [27]	−11.44	−25.47, 2.59	0.110	−8.81	−15.36, −2.26	0.008	−14.25	−24.13, −4.37	0.005	1.40	−1.65, 4.46	0.367
Bumrungpert, A. (2019) ^a^, [28]	−15.88	−32.11, 0.35	0.055	−12.00	−20.22, −3.78	0.004	−15.76	−26.13, −5.4	0.003	1.79	−1.1, 4.68	0.225
Bumrungpert, A. (2019) ^b^, [28]	−17.12	−32.65, −1.6	0.031	−11.82	−19.94, −3.69	0.004	−15.07	−25.28, −4.87	0.004	2.20	−0.56, 4.96	0.118
Bumrungpert, A. (2019) ^c^, [28]	−15.21	−30.87, 0.45	0.057	−11.60	−19.69, −3.51	0.005	−15.46	−25.77, −5.16	0.003	1.87	−1.00, 4.74	0.202
Borresen, E.C. (2017), [29]	−19.29	−32.93, −5.65	0.006	−13.67	−21.44, −5.89	0.001	−17.16	−27.53, −6.79	0.001	1.66	−1.36, 4.69	0.281
Borresen, E.C. (2016), [30]	−14.92	−30.05, 0.22	0.053	−12.22	−20.12, −4.31	0.002	−9.98	−16.65, −3.3	0.003	1.86	−0.95, 4.67	0.194
Salar, A. (2016), [31]	−10.40	−23.87, 3.06	0.130	−10.09	−17.54, −2.64	0.008	−15.79	−26.22, −5.37	0.003	2.23	−0.65, 5.11	0.128
Lai, M.-H. (2012), [32]	−16.84	−31.56, −2.12	0.025	−12.27	−20.55, −3.99	0.004	−16.02	−26.38, −5.65	0.002	1.87	−1.05, 4.79	0.210
Cheng, H.-H. (2010), [33]	−13.70	−28.62, 1.21	0.072	−11.19	−18.86, 3.53	0.004	−14.32	−24.09, −4.55	0.004	2.15	−0.62, 4.92	0.129
Gerhardt, A.L. (1998), [34]	−14.44	−29.64, 0.75	0.062	−10.88	−18.81, −2.95	0.007	−14.87	−25.12, −4.62	0.004	1.74	−1.18, 4.66	0.243
Wijayanthie, N. (2019), [35]	−17.77	−33.12, −2.42	0.023	-	-	-	-	-	-	-	-	-
Choi, J.-Y. (2014), [36]	-	-	-	−13.20	−21.66, −4.73	0.002	-	-	-	-	-	-
Hegsted, M. (1993), [37]	−14.33	−30.49, 1.82	0.082	−13.86	−21.71, −6.02	0.001	−17.42	−26.88, −7.96	0.000	0.58	−1.57, 2.74	0.596

CI, confidence interval; TGs, Triglycerides; TC, Total Cholesterol; LDL-C, Low-Density Lipoprotein Cholesterol; HDL-C, High-Density Lipoprotein Cholesterol; ^a, b, c^ represent rice bran containing different levels of gamma-oryzanol: a = 4000 ppm, b = 8000 ppm, c = 11,000 ppm.

**Table 3:** Subgroup analysis of interventions on total cholesterol and low-density lipoprotein cholesterol.

Subgroup	TC			LDL-C		
	No. of Studies	Effect Size (95% CI)	*I* ^2^	No. of Studies	Effect Size (95% CI)	*I* ^2^
Study design						
Parallel	11	−13.86 (−21.71, −6.02) *	31.1	10	−17.42 (−26.88, −7.96) *	44.3
Cross-over	1	−0.39 (−9.04, 8.26)	0.00	1	−0.38 (−6.55, 5.79)	0.00
Health status						
Healthy	1	−3.48 (−18.30, 11.34)	0.00	-	-	-
Hyperlipidemia	6	−7.12 (−15.54, 1.30)	33.0	6	−7.25 (−14.97, 0.47)	29.6
T2DM	3	−19.85 (−33.57, −6.12) *	0.00	3	−13.77 (−26.85, −0.68)	0.00
Others	2	−25.31 (−57.54, 6.92)	49.8	2	−50.83 (−106.41, 4.49)	83.7
Intervention						
Rice bran oil	6	−19.24 (−28.16, −10.33) *	0.00	6	−14.55 (−23.24, −5.87) *	0.00
Rice bran	5	−5.65 (−16.70, 5.41)	40.3	5	−19.34 (−38.86, 0.18)	81.8
Fermented rice bran	1	−3.48 (−18.30, −11.34)	0.00	-	-	-
Dose						
<30 g(mL)	4	−3.60 (−13.43, 6.22)	7.35	3	−8.45 (−22.89, 5.98)	18.4
≥30 g(mL)	8	−15.94 (−26.22, −5.67) *	52.7	8	−17.72 (−29.86, −5.59) *	71.5
Duration						
≤4 weeks	7	−7.80 (−16.57, 0.97)	34.4	7	−13.70 (−26.42, −0.98) *	70.9
>4 weeks	5	−17.45 (−30.28, −4.62) *	44.9	4	−18.81 (−29.88, −7.73) *	0.00
Country						
Asia	8	−16.24 (−24.56, −7.91) *	16.0	7	−15.54 (−23.88, −7.20) *	0.00
USA	4	−3.61 (−14.11, 6.90)	37.5	4	−18.11 (−40.17, 3.95)	84.8
Gender						
Both	10	−5.86 (−11.43, −0.30) *	2.89	9	−14.91 (−25.91, −3.91) *	67.2
Men	1	−38.00 (−61.47, −14.53) *	0.00	1	−24.69 (−47.69, −1.69) *	0.00
Women	1	−27.71 (−48.76, −6.67) *	0.00	1	−11.81 (−31.59, 7.97)	0.00
Age						
<18	1	5.00 (−11.02, 21.02)	0.00	1	1.00 (−18.03, 20.03)	0.00
≥18	11	−13.67 (−21.45, −5.89) *	39.8	10	−17.16 (−27.53, −6.79) *	65.7

TC, Total Cholesterol; LDL-C, Low-Density Lipoprotein Cholesterol; * *p* < 0.05.

## Data Availability

The data presented in this study are available in the article.
